# Supplementary material for: Near-infrared-II photoacoustic imaging and photo-triggered synergistic treatment of thrombosis via fibrin-specific homopolymer nanoparticles
Source: Nat Commun. 2023 Oct 28;14:6881. doi: 10.1038/s41467-023-42691-8 (PMC10613240; doi:10.1038/s41467-023-42691-8)
Supplement: Supplementary file 1 — Supplementary information [file 41467_2023_42691_MOESM1_ESM.pdf]

Supplementary information for

Near-infrared-II photoacoustic imaging and photo-triggered synergistic treatment of thrombosis via fibrin-specific homopolymer nanoparticles

Jianwen Song,[1] Xiaoying Kang,[1] Lu Wang,[2] Dan Ding,[1] Deling Kong,[1]* Wen Li[2]* & Ji Qi[1]*

[1]State Key Laboratory of Medicinal Chemical Biology, Key Laboratory of Bioactive Materials, Ministry of Education, Frontiers Science Center for Cell Responses, and College of Life Sciences, Nankai University, Tianjin 300071, China.

[2]Tianjin Key Laboratory of Biomedical Materials and Key Laboratory of Biomaterials and Nanotechnology for Cancer Immunotherapy, Institute of Biomedical Engineering, Chinese Academy of Medical Sciences and Peking Union Medical College, Tianjin 300192, China.

*Correspondence should be addressed to D.K. (Email: kongdeling@nankai.edu.cn), W.L. (Email: liwen@bme.pumc.edu.cn) and J.Q. (Email: qiji@nankai.edu.cn).

Supplementary Figures

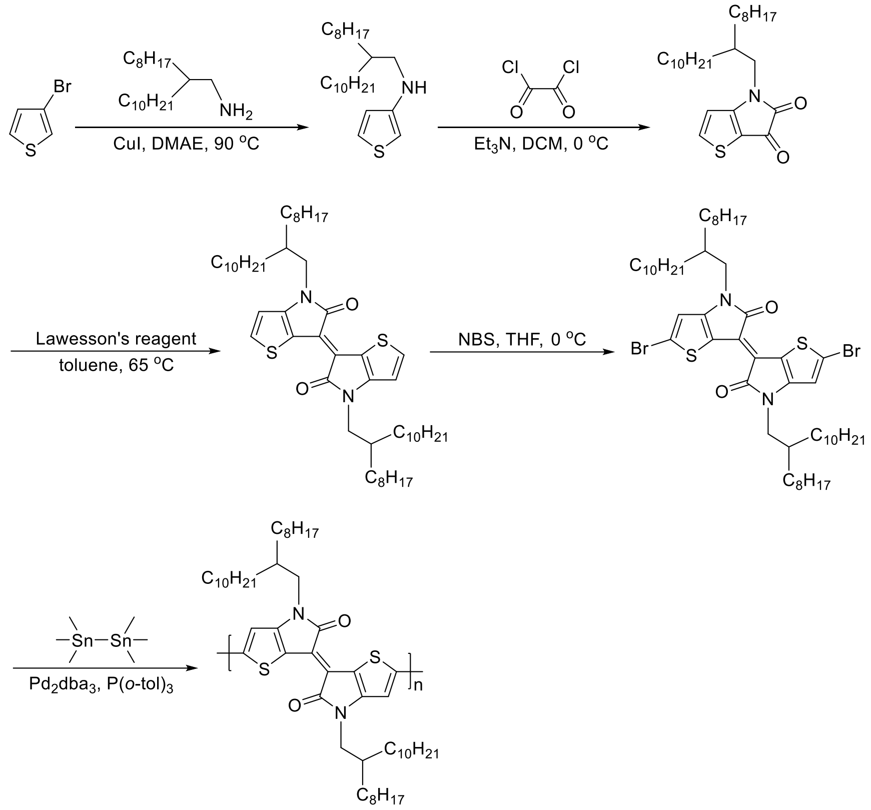

Supplementary Fig. 1. Synthetic route to PTIIG.

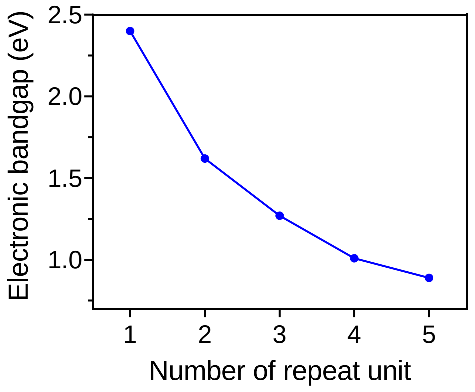

Supplementary Fig. 2. The relationship between the numbers of repeat units and corresponding electronic bandgaps of PTIIG. Source data are provided as a Source Data file.

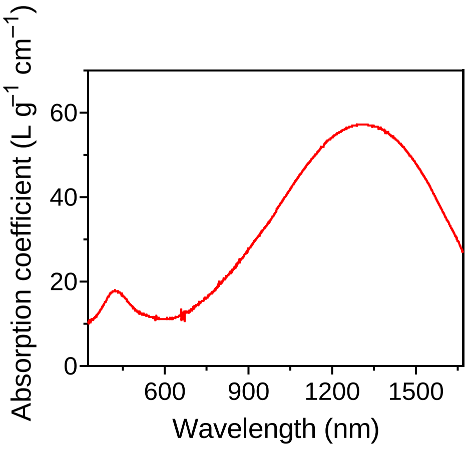

Supplementary Fig. 3. Absorption spectrum of PTIIG in THF. Experiment was repeated three times independently with similar results. Source data are provided as a Source Data file.

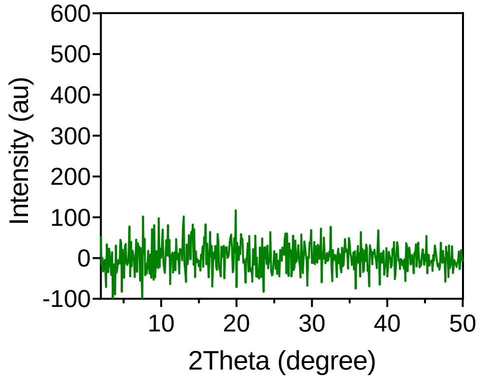

Supplementary Fig. 4. X-ray diffraction diagram of PTIIG film. Experiment was repeated three times independently with similar results. Source data are provided as a Source Data file.

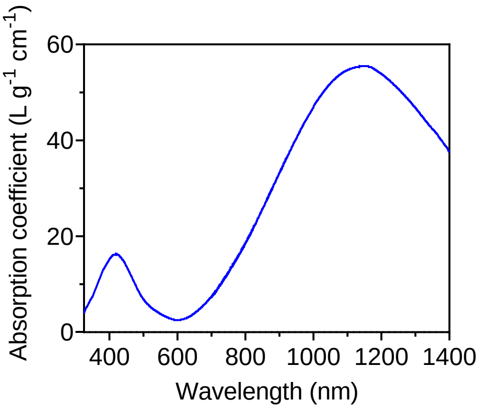

Supplementary Fig. 5. Absorption spectrum of SP NPs in PBS (the concentration is based on PTIIG polymer). Experiment was repeated three times independently with similar results. Source data are provided as a Source Data file.

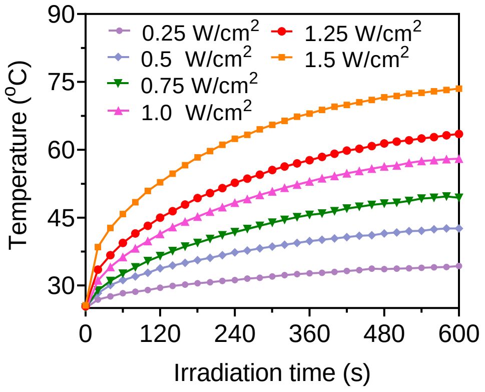

Supplementary Fig. 6. The photothermal heating curves of SP NPs (100 μg mL^{-1}) under the irradiation of 1064 nm light with different power densities as indicated. Experiment was repeated three times independently with similar results. Source data are provided as a Source Data file.

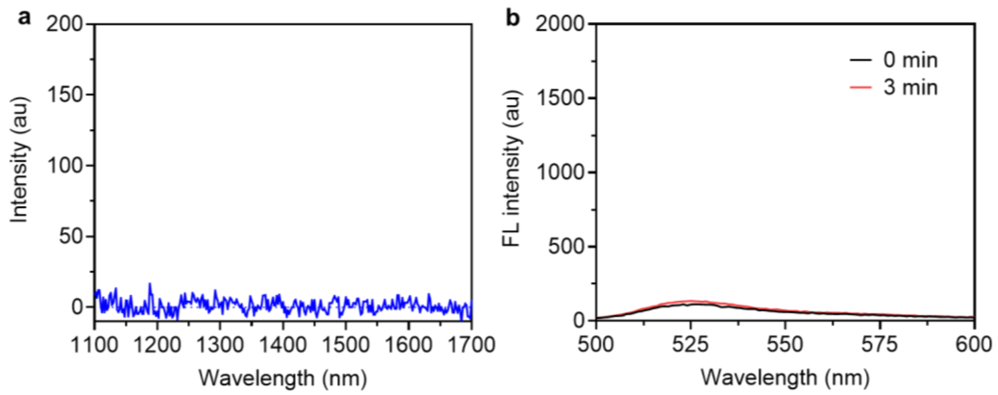

Supplementary Fig. 7. a The photoluminescence spectrum of SP NPs with the excitation of 1064 nm light. **b** The ROS production of SP NPs under light irradiation measured using DCFH-DA as the ROS indicator. Experiment was repeated three times independently with similar results. Source data are provided as a Source Data file.

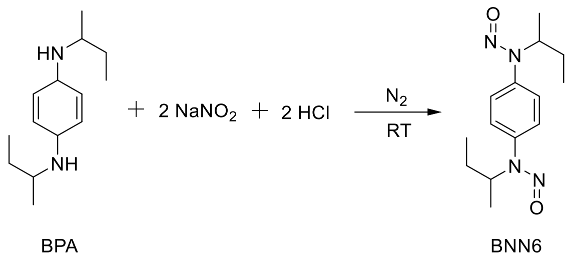

Supplementary Fig. 8. Synthetic route to BNN6.

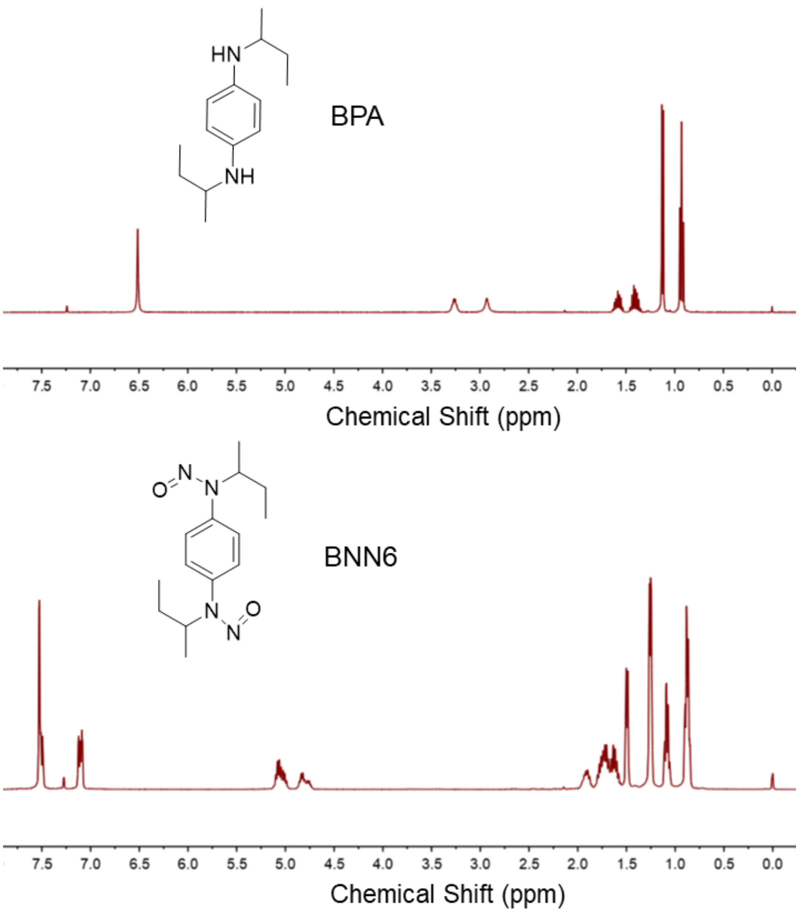

Supplementary Fig. 9. ^{1}H NMR spectra of BPA and BNN6 (400 MHz, $CDCl_3$).

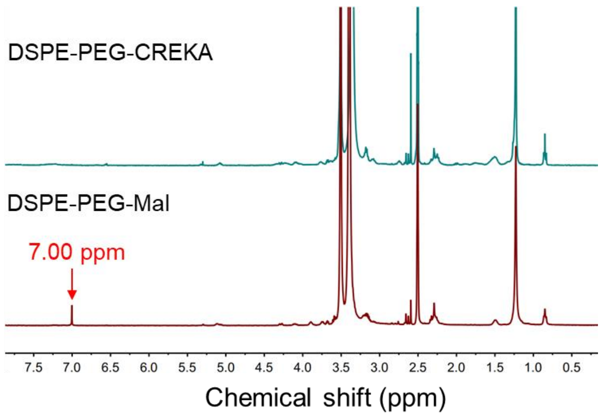

Supplementary Fig. 10. ^{1}H NMR spectra of DSPE-PEG-Mal and DSPE-PEG-CREKA (400 MHz, DMSO-d_6). The disappearance of the chemical shift of maleimide group at 7.0 ppm indicated the successful conjugation of CREKA to DSPE-PEG-Mal.[1,2]

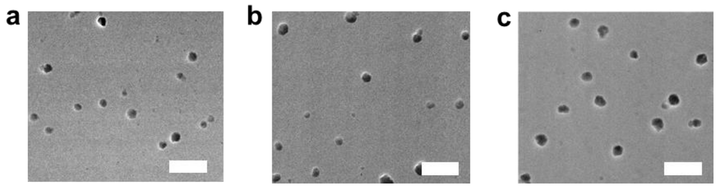

Supplementary Fig. 11. Representative TEM images of (a) SP NPs, (b) B@SP NPs and (c) B@SP−C NPs. Scale bars = 500 nm. Experiment was repeated three times independently with similar results.

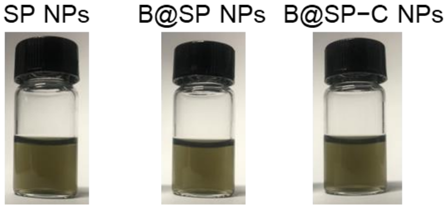

Supplementary Fig. 12. Photographs of the solution of SP NPs, B@SP NPs, and B@SP−C NPs.

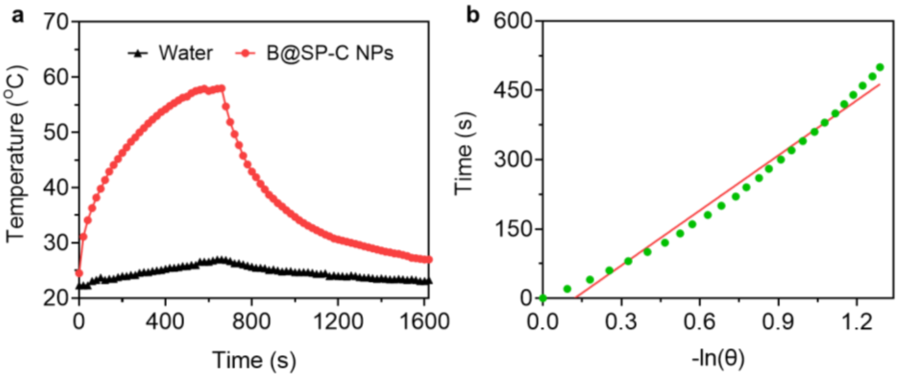

Supplementary Fig. 13. a The photothermal behavior of water and B@SP−C NPs, which were irradiated with 1064 nm light for 11 min, then the laser was removed, and the samples were naturally cooling down. **b** Plots of irradiation time versus -ln(θ) for B@SP−C NPs. The slope indicates its system time constant (τ_s). The photothermal conversion efficiency could be calculated.[3,4] Experiment was repeated three times independently with similar results. Source data are provided as a Source Data file.

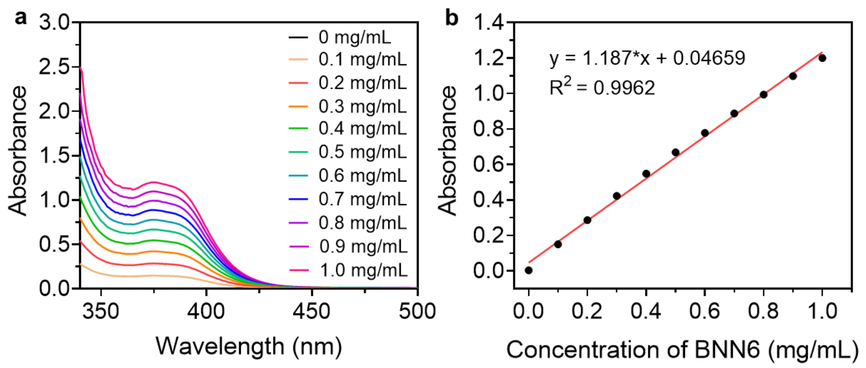

Supplementary Fig. 14. **a** UV-vis absorption spectra of BNN6 solution at different concentrations, and **b** the absorbance at 375 nm versus the concentration of BNN6 was plotted. Experiment was repeated three times independently with similar results. Source data are provided as a Source Data file.

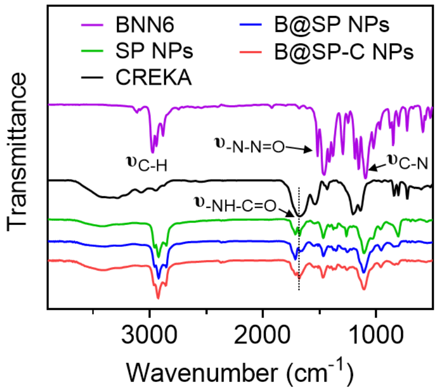

Supplementary Fig. 15. Fourier transform infrared spectroscopy (FT-IR) spectra of BNN6, CREAK, SP NPs, B@SP NPs and B@SP−C NPs. Source data are provided as a Source Data file.

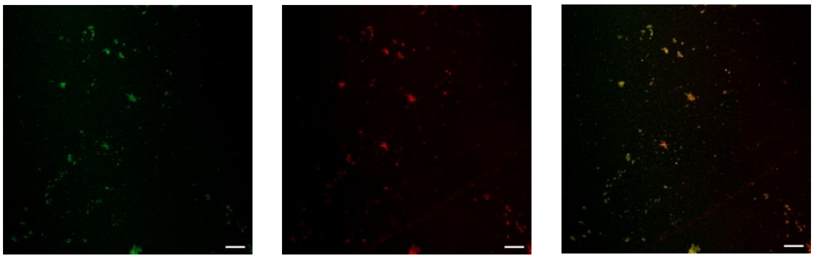

Supplementary Fig. 16. The colocalization of Dil-loaded B@SP−C NPs (red fluorescence) with FITC-labeled fibrin (green fluorescence) indicating the binding between them. Dil is 1,1-dioctadecyl-3,3,3’,3’-tetramethylindocarbocyanine perchlorate and FITC is fluorescein isothiocyanate. Scale bars = 50 μm. Experiment was repeated three times independently with similar results.

Batch	Size (nm)	PDI	Encapsulation efficiency	
			PTIIG	BNN6
1	166.5±3.5	0.19±0.03	59.4%	42.6%
2	168.3±3.5	0.18±0.01	62.1%	40.1%
3	167.8±4.1	0.19±0.01	60.9%	41.5%
4	169.4±4.7	0.2±0.02	62.5%	39.5%
5	167.4±2.8	0.19±0.02	58.9%	40.9%

Supplementary Fig. 17. B@SP−C NPs prepared from different batches were analyzed for their hydrodynamic diameters, PDI and the encapsulation efficiencies of PTIIG and BNN6.

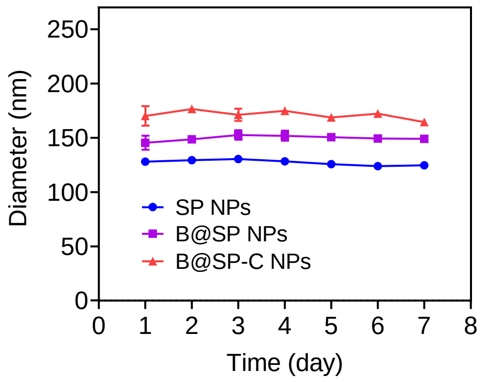

Supplementary Fig. 18. The colloidal stability of SP NPs, B@SP NPs, and B@SP−C NPs during one-week storage measured by DLS. Data were presented as mean ± SD (*n* = 3 independent experiments). Source data are provided as a Source Data file.

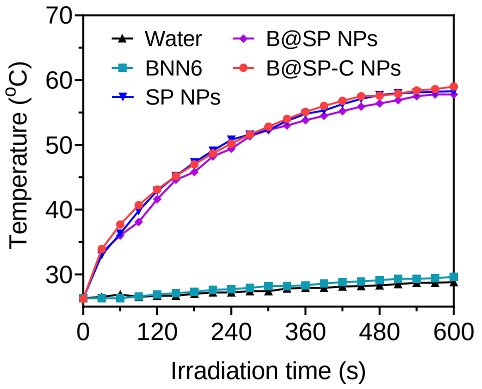

Supplementary Fig. 19. The photothermal heating curves of water, BNN6, SP NPs, B@SP NPs, and B@SP−C NPs under the irradiation of 1064 nm laser for different time. Experiment was repeated three times independently with similar results. Source data are provided as a Source Data file.

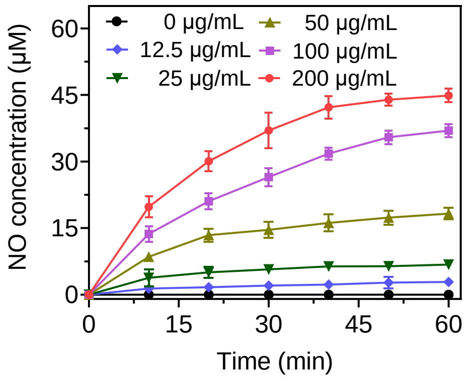

Supplementary Fig. 20. The amount of NO released from different concentrations of B@SP−C NPs upon 1064 light (1 W cm^{-2}) excitation. Data were presented as mean ± SD (n = 3 independent experiments). Source data are provided as a Source Data file.

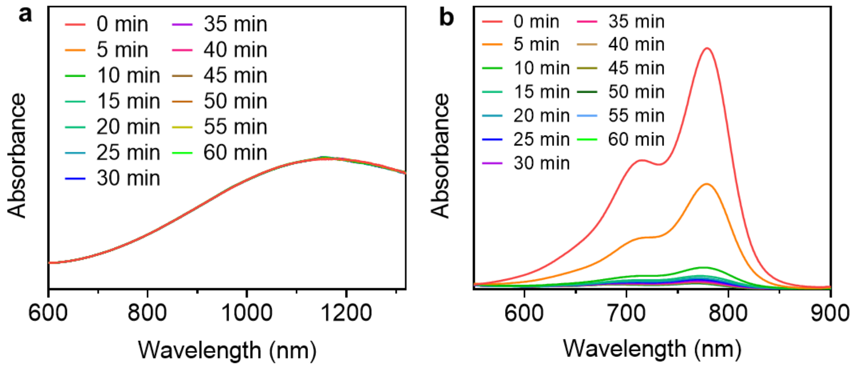

Supplementary Fig. 21. Absorption spectra of (a) B@SP−C NPs solution after 1064 nm NIR-II laser (1.0 W cm^{-2}) irradiation and (b) ICG solution after 808 nm laser (1.0 W cm^{-2}) irradiation for different time. Experiment was repeated three times independently with similar results. Source data are provided as a Source Data file.

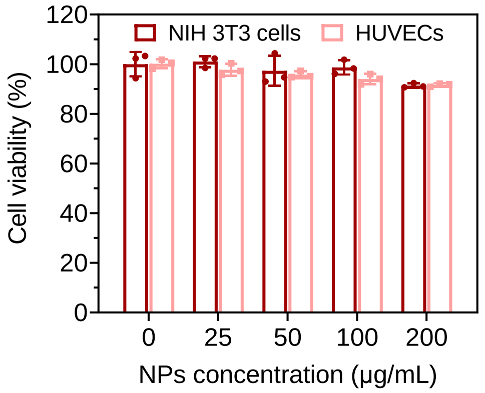

Supplementary Fig. 22. The relative viabilities of NIH 3T3 cells and HUVEC cells after incubation with various concentrations of B@SP−C NPs for 24 h measured by MTT assay. Data were presented as mean ± SD (*n* = 3 independent experiments). Source data are provided as a Source Data file.

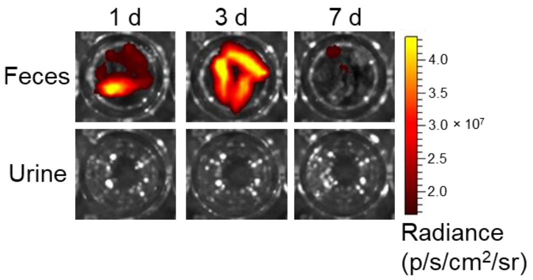

Supplementary Fig. 23. Fluorescent images of the collected feces and urine at various time points after intravenous injection of B@SP−C NPs (loaded with DiR) into mice. Experiment was repeated three times independently with similar results.

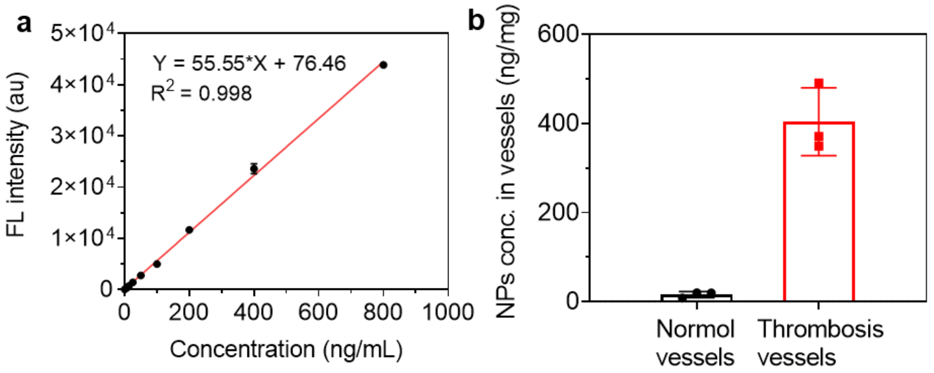

Supplementary Fig. 24. a The relationship of fluorescence intensity and different concentrations of DiR in methanol. Data are presented as mean ± SD (n = 3 independent experiments). **b** The concentrations of B@SP−C NPs (loaded with DiR) in normal vessel and thrombosis vessel of mice calculated from the standard curve in **a**. Data are presented as mean ± SD (n = 3 mice). Source data are provided as a Source Data file.

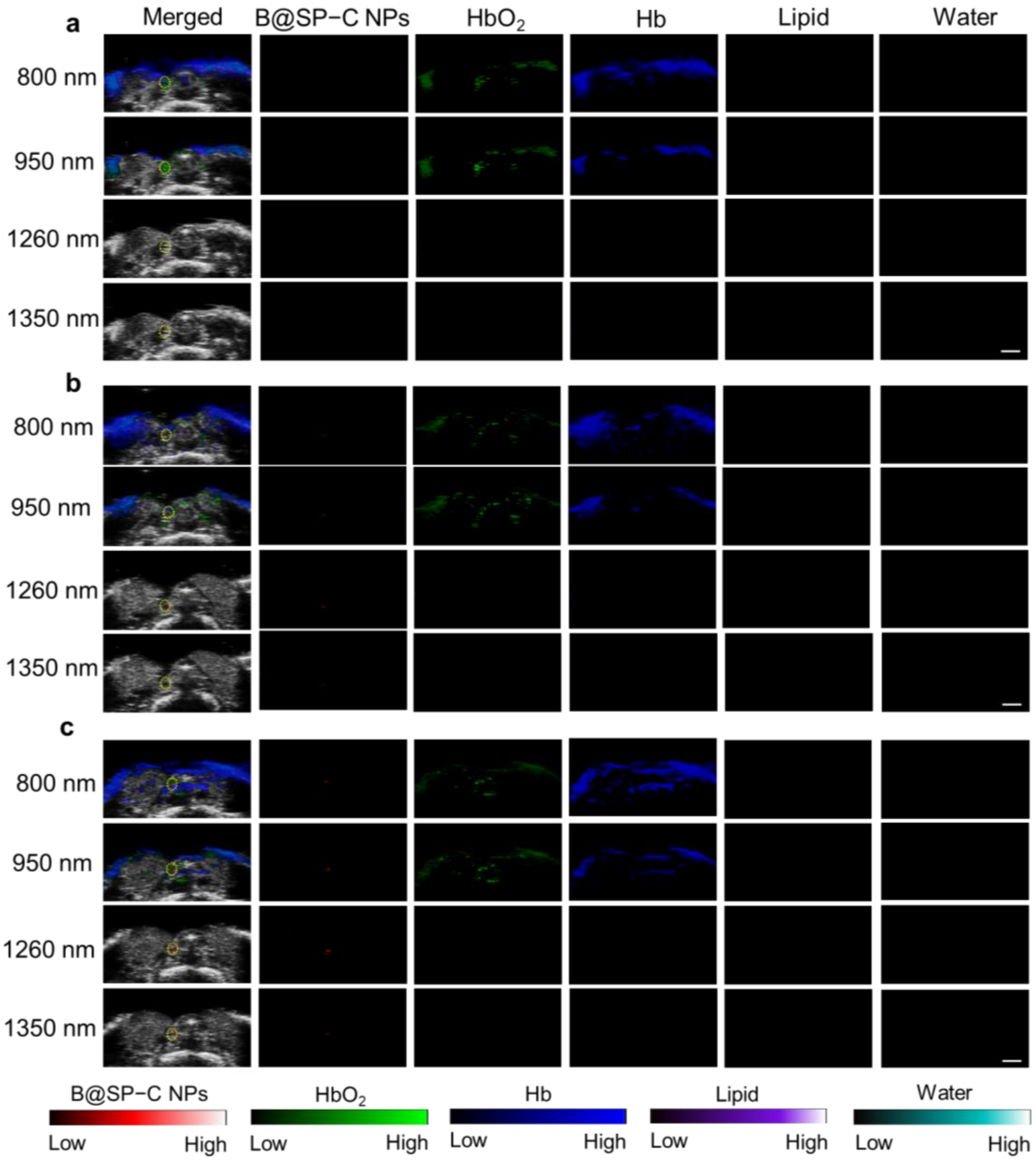

Supplementary Fig. 25. The merged and de-merged spectral unmixing images of the in vivo NIR-II PA imaging of thrombus site with the excitation of 800, 950, 1260 and 1350 nm laser after the treatments of (a) PBS, (b) B@SP NPs and (c) B@SP−C NPs, respectively. Yellow circle indicated the thrombotic artery. Scale bars = 2 mm.

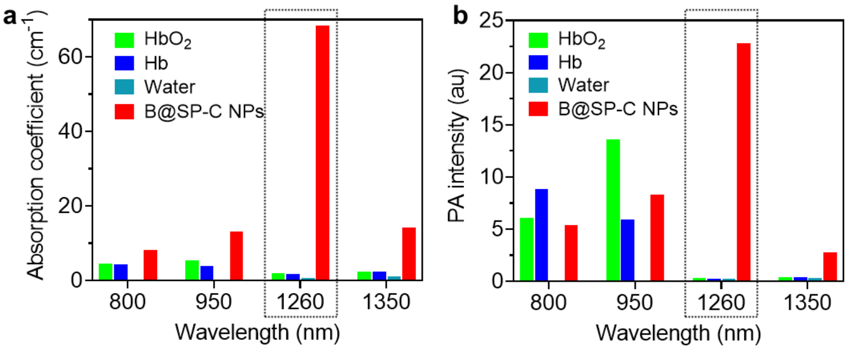

Fig. 26. (a) The comparison of the absorption coefficient and (b) in vivo PA intensity of different contrasts at various wavelengths. The absorption coefficients of hemoglobin and water aligned well with those reported in the literatures.[5,6] The excitation wavelength of 1260 nm (indicated by dotted squares) was finally chosen for in vivo PA imaging. Source data are provided as a Source Data file.

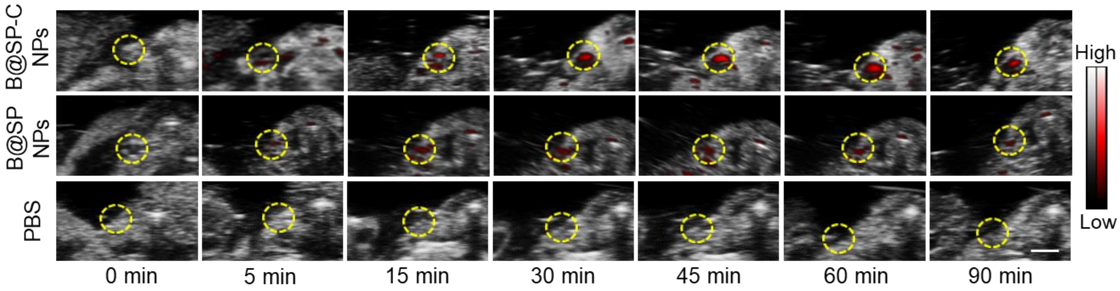

Supplementary Fig. 27. The NIR-II PA/US merged images in Fig. 6c were enlarged to enable better comparison. Yellow circle indicated the thrombotic artery. The grey and red bars represent ultrasound and PA signals, respectively. Scale bar = 1 mm.

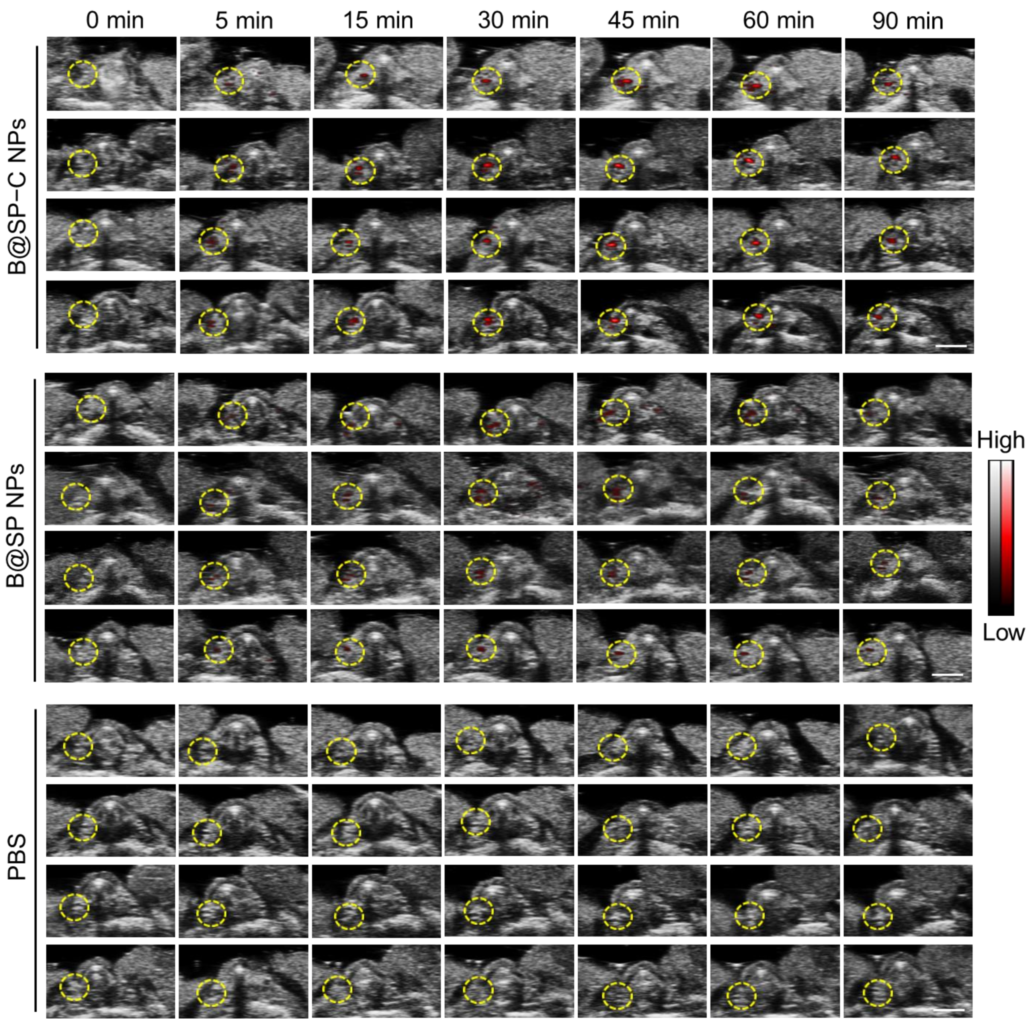

Supplementary Fig. 28. The other four mice results for the in vivo NIR-II PA/US merged images of the thrombotic artery at different time points as indicated after the mice were treated with PBS, B@SP NPs, or B@SP−C NPs, respectively. The PA imaging was conducted at the excitation of 1260 nm light. Yellow circle indicated the thrombotic artery. The grey and red bars represent ultrasound and PA signals, respectively. Scale bars = 2 mm.

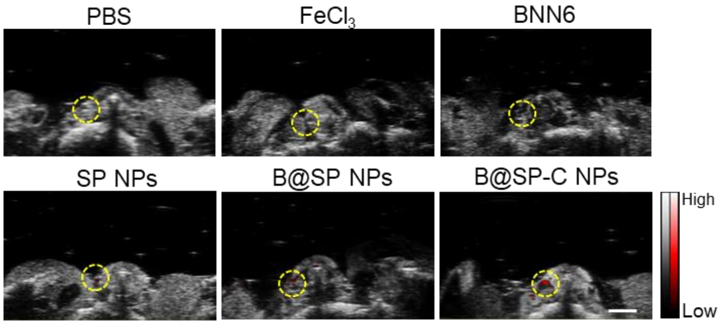

Supplementary Fig. 29. Representative in vivo PA/US merged images of thrombus-bearing mice after various treatments under the excitation of 1260 nm laser. Yellow circle indicated the thrombotic artery. The grey and red bars represent ultrasound and PA signals, respectively. Scale bar = 2 mm.

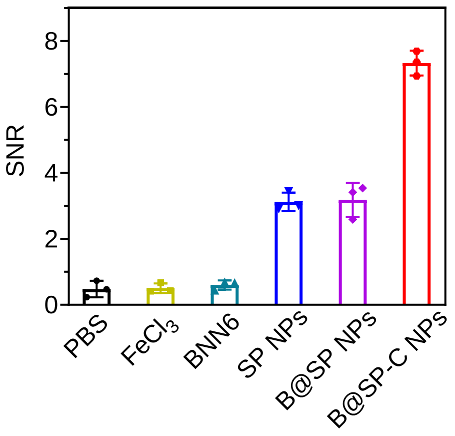

Supplementary Fig. 30. SNR of the PA images of thrombus-bearing mice under the excitation of 1260 nm laser at 45 min after various treatments. Data are presented as mean ± SD ($n = 3$ mice). Source data are provided as a Source Data file.

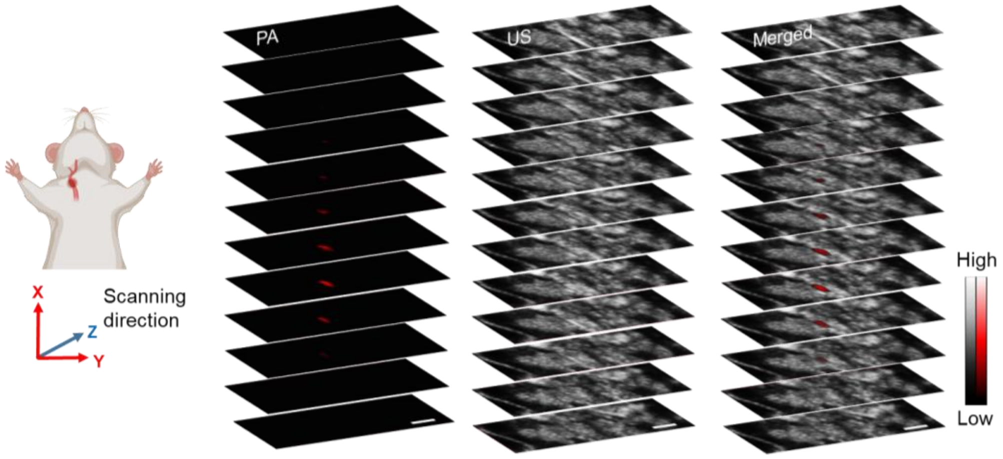

Supplementary Fig. 31. The thrombus region of mice injected with B@SP-C NPs was scanned in the Z direction to acquire multiple slices of PA images. The grey and red bars represent ultrasound and PA signals, respectively. Slide step = 0.05 mm, Scale bars = 2 mm. The illustration was created with the help of BioRender.com.

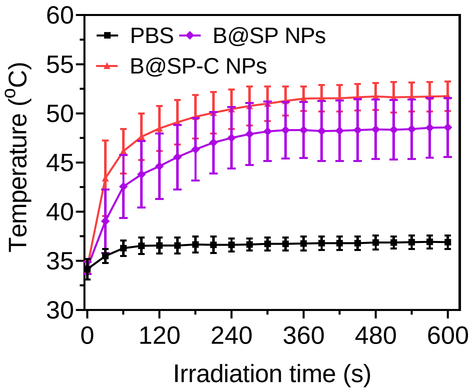

Supplementary Fig. 32. The photothermal heating curves of thrombus-bearing mice under 1064 nm (1 W cm^{-2}) light irradiation at 45 min after intravenous injection of PBS, B@SP NPs or B@SP−C NPs. Data were presented as mean ± SD (n = 3 mice). Source data are provided as a Source Data file.

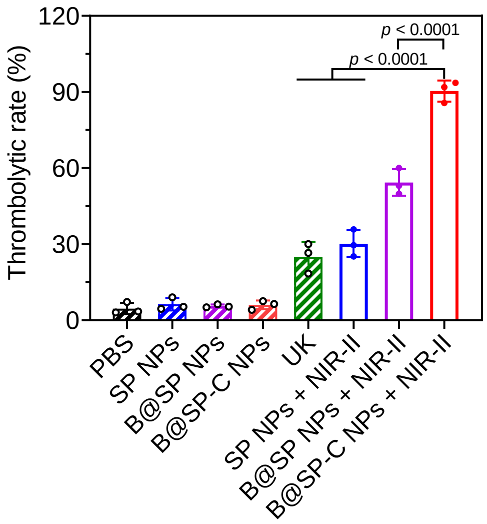

Supplementary Fig. 33. Evaluation of thrombolytic efficiency according to the blood vessel sections from mice after different treatments. Data were presented as mean ± SD ($n = 3$ mice). P values were calculated using one-way ANOVA for multiple comparisons. Source data are provided as a Source Data file.

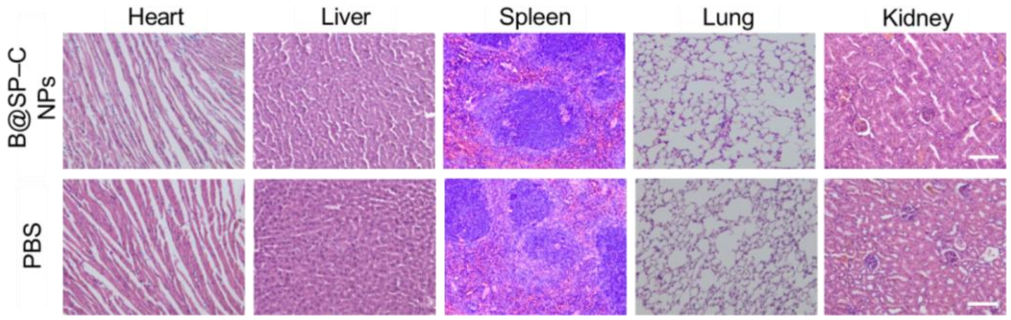

Supplementary Fig. 34. H&E staining of tissue sections from major organs of mice injected with PBS or B@SP−C NPs. Mice were euthanized at day 7 after treatment, and the major organs were collected for H&E staining. Scale bars = 100 μm. Experiment was repeated three times independently with similar results.

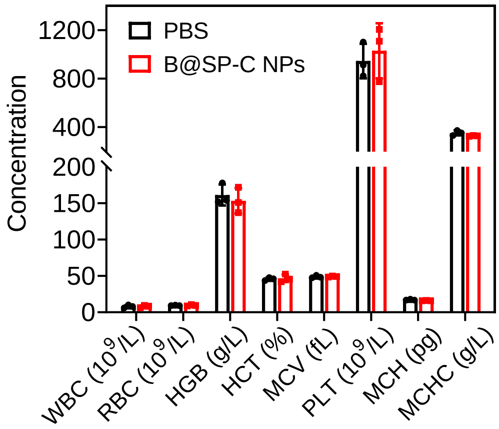

Supplementary Fig. 35. The routine blood test data of the mice with different treatments. Data were presented as mean ± SD (n = 3 mice). Source data are provided as a Source Data file.

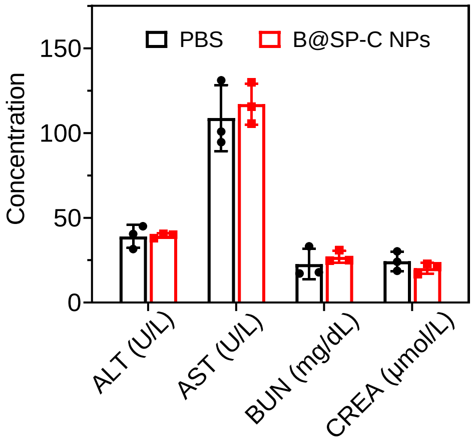

Supplementary Fig. 36. The hepatorenal indicator analysis of the mice with different treatments. Data were presented as mean ± SD (n = 3 mice). Source data are provided as a Source Data file.

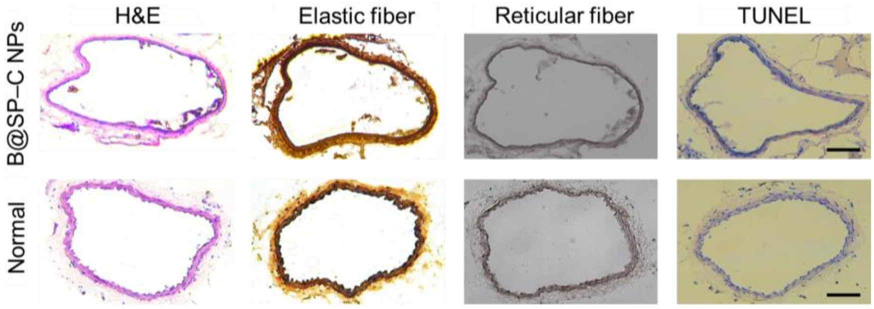

Supplementary Fig. 37. Cross-sectional histology analysis of normal mice blood vessels and the 1064 nm NIR-II laser (1 W cm^{-2}) irradiation-treated blood vessels (stained with H&E, elastic fiber staining, reticular fiber staining and TUNEL). Scale bars = 100 µm. Experiment was repeated three times independently with similar results.

References

1. Zhao, Y. et al. Biomimetic fibrin-targeted and H_2O_2-responsive nanocarriers for thrombus therapy. *Nano Today* **35**, 100986 (2020).

2. Wu, Q. et al. Modification of adipose mesenchymal stem cells-derived small extracellular vesicles with fibrin-targeting peptide CREKA for enhanced bone repair. *Bioact. Mater.* **20**, 208 (2023).

3. Li, J. et al. Second near-infrared photothermal semiconducting polymer nanoadjuvant for enhanced cancer immunotherapy. *Adv. Mater.* **33**, 2003458 (2021).

4. Liu, R. et al. Advances of nanoparticles as drug delivery systems for disease diagnosis and treatment. *Chin. Chem. Lett.* **34**, 107518 (2022).

5. Weber, J., Beard, P. C. & Bohndiek, S. E. Contrast agents for molecular photoacoustic imaging. *Nat. Methods* **13**, 639 (2016).

6. Nachabé, R. et al. Effect of bile absorption coefficients on the estimation of liver tissue optical properties and related implications in discriminating healthy and tumorous samples. *Biomed. Opt. Express* **2**, 600 (2011).

7. Grimme, S., Antony, J., Ehrlich, S. & Krieg, H. A consistent and accurate ab initio parametrization of density functional dispersion correction (DFT-D) for the 94 elements H-Pu. *J. Chem. Phys.* **132**, 154104 (2010).
