# Supplementary material for: Near-infrared-II photoacoustic imaging and photo-triggered synergistic treatment of thrombosis via fibrin-specific homopolymer nanoparticles
Source: Nat Commun. 2023 Oct 28;14:6881. doi: 10.1038/s41467-023-42691-8 (PMC10613240; doi:10.1038/s41467-023-42691-8)
Supplement: Supplementary file 3 — Description of Additional Supplementary Files [file 41467_2023_42691_MOESM3_ESM.pdf]

Description of Additional Supplementary Files

Supplementary dataset 1: Cartesian coordinates of the oligomers with different numbers of repeat units (TIIG1–5) calculated by the DFT, B3LYP/6-31G(d), Gaussian 09 program.
