# Supplementary material for: Near-infrared-II photoacoustic imaging and photo-triggered synergistic treatment of thrombosis via fibrin-specific homopolymer nanoparticles
Source: Nat Commun. 2023 Oct 28;14:6881. doi: 10.1038/s41467-023-42691-8 (PMC10613240; doi:10.1038/s41467-023-42691-8)
Supplement: Supplementary file 5 — Reporting Summary [file 41467_2023_42691_MOESM5_ESM.pdf]

nature portfolio

Corresponding author(s): Deling Kong, Wen Li, Ji Qi

Last updated by author(s): Sep 30, 2023

Reporting Summary

Nature Portfolio wishes to improve the reproducibility of the work that we publish. This form provides structure for consistency and transparency in reporting. For further information on Nature Portfolio policies, see our Editorial Policies and the Editorial Policy Checklist.

Statistics

For all statistical analyses, confirm that the following items are present in the figure legend, table legend, main text, or Methods section.

n/a	Confirmed	
☐	☒	The exact sample size (*n*) for each experimental group/condition, given as a discrete number and unit of measurement
☐	☒	A statement on whether measurements were taken from distinct samples or whether the same sample was measured repeatedly
☐	☒	The statistical test(s) used AND whether they are one- or two-sided<br>*Only common tests should be described solely by name; describe more complex techniques in the Methods section.*
☒	☐	A description of all covariates tested
☒	☐	A description of any assumptions or corrections, such as tests of normality and adjustment for multiple comparisons
☐	☒	A full description of the statistical parameters including central tendency (e.g. means) or other basic estimates (e.g. regression coefficient) AND variation (e.g. standard deviation) or associated estimates of uncertainty (e.g. confidence intervals)
☐	☒	For null hypothesis testing, the test statistic (e.g. *F*, *t*, *r*) with confidence intervals, effect sizes, degrees of freedom and *P* value noted<br>*Give P values as exact values whenever suitable.*
☒	☐	For Bayesian analysis, information on the choice of priors and Markov chain Monte Carlo settings
☒	☐	For hierarchical and complex designs, identification of the appropriate level for tests and full reporting of outcomes
☒	☐	Estimates of effect sizes (e.g. Cohen's *d*, Pearson's *r*), indicating how they were calculated

Our web collection on statistics for biologists contains articles on many of the points above.

Software and code

Policy information about availability of computer code

Data collection	DLS: Malvern Zetasizer Nano-ZS; TEM: FEI Talos L120C G2; FT-IR: TENSOR 37; UV-vis: Shimadzu UV-3600 plus spectrometer; FL: Edinburgh FLS1000 spectrometer; Lasers: Changchun Laser Optical 1064; DFT: Gaussian 09 program package (revision D. 01) at the level of B3LYP/6-31G*; Thermal imaging camera: FLK-Ti200; Confocal imaging: LSM 800 with Airyscan; In-vivo imaging: PerkinElmer, IVIS Spectrum; PA imaging: Vevo® LAZR-X, Fuji VisualSonics; Microplate reader: spark spectrometer; The laser speckle imaging system: RWD, RFLSI III; The Doppler ultrasound imaging system: VEVO, Vevo2100; Digital microscope: Leica.
Data analysis	Graphpad Prism (version 8.0.2); Image J2x (version 2.1.4.7); PA image: Vevo LAB 5.6.1; Gauss View 5.0; ZEN 2012 (version 1.1.2.0); NMR: MestReNova (Version: 6.1.0-6224); GaussView (version 5.0); DLS: Zetasizer Software 7.12 In-vivo imaging: Living Image 4.2; ChemDraw 20.1; The laser speckle imaging: LSCI V1.0.0.

For manuscripts utilizing custom algorithms or software that are central to the research but not yet described in published literature, software must be made available to editors and reviewers. We strongly encourage code deposition in a community repository (e.g. GitHub). See the Nature Portfolio guidelines for submitting code & software for further information.

Data

Policy information about availability of data

All manuscripts must include a data availability statement. This statement should provide the following information, where applicable:

- Accession codes, unique identifiers, or web links for publicly available datasets
- A description of any restrictions on data availability
- For clinical datasets or third party data, please ensure that the statement adheres to our policy

All data are available within the Article, Supplementary Information or Source Data file. Source data are provided with this paper.

Research involving human participants, their data, or biological material

Policy information about studies with human participants or human data. See also policy information about sex, gender (identity/presentation), and sexual orientation and race, ethnicity and racism.

Reporting on sex and gender	not applicable
Reporting on race, ethnicity, or other socially relevant groupings	not applicable
Population characteristics	not applicable
Recruitment	not applicable
Ethics oversight	not applicable

Note that full information on the approval of the study protocol must also be provided in the manuscript.

Field-specific reporting

Please select the one below that is the best fit for your research. If you are not sure, read the appropriate sections before making your selection.

☒ Life sciences ☐ Behavioural & social sciences ☐ Ecological, evolutionary & environmental sciences

For a reference copy of the document with all sections, see nature.com/documents/nr-reporting-summary-flat.pdf

Life sciences study design

All studies must disclose on these points even when the disclosure is negative.

Sample size	Sample size estimates have been performed on previous experience to obtain statistical significance and reproducibility. For in vitro experiments such as photoacoustic experiments and thrombolytic experiments, at least three samples were used per group for minimal statistics requirements. For in vivo studies, the sample size was determined to be sufficient to obtain the statistical difference between groups.
Data exclusions	No data were excluded from the analyses.
Replication	The experimental results are repeatable in our experiments if all the methods and material are in compliance with what we state in the Methods section. All experiments underlying main conclusions of this study have been successfully replicated multiple times and corroborated by several models. All in vitro and in vivo experiments were carried out at least three times independently with the similar results.
Randomization	All samples/organisms were randomly allocated into experimental groups.
Blinding	The investigators were blinded to group allocation during data collection and analysis.

Reporting for specific materials, systems and methods

We require information from authors about some types of materials, experimental systems and methods used in many studies. Here, indicate whether each material, system or method listed is relevant to your study. If you are not sure if a list item applies to your research, read the appropriate section before selecting a response.

Materials & experimental systems

n/a	Involved in the study	
☒	☐	Antibodies
☐	☒	Eukaryotic cell lines
☒	☐	Palaeontology and archaeology
☐	☒	Animals and other organisms
☒	☐	Clinical data
☒	☐	Dual use research of concern
☒	☐	Plants

Methods

n/a	Involved in the study	
☒	☐	ChIP-seq
☒	☐	Flow cytometry
☒	☐	MRI-based neuroimaging

Eukaryotic cell lines

Policy information about cell lines and Sex and Gender in Research

Cell line source(s)	NIH 3T3 were purchased from Cell Bank of Shanghai, Chinese Academy of Sciences (Shanghai, China) with Catalog #: GNM 6; HUVECs were purchased from Cyagen Biosciences (Guangzhou, China) Inc. with Catalog #: HUVEC-20001.
Authentication	The cell lines were certified by the manufacturers (surface markers, morphology).
Mycoplasma contamination	Cells were routinely screened for free of Mycoplasma contaminations. All cell lines are Mycoplasma negative with this study.
Commonly misidentified lines (See ICLAC register)	None of the cell lines used in this study is commonly misidentified cell line.

Animals and other research organisms

Policy information about studies involving animals; ARRIVE guidelines recommended for reporting animal research, and Sex and Gender in Research

Laboratory animals	Male C57BL/6 mice (6 weeks old) were purchased from Laboratory Animal Center of the Academy of Military Medical Sciences (Beijing, China). All procedures involving animal were conducted in accordance with the guidelines set by the Tianjin Committee of Use and Care of Laboratory Animals, and approved by the Animal Ethics Committee of Nankai University. All mice were cultured in suitable temperature and humidity environment (25 ℃, suitable humidity (typically 50 %), 12 hour dark/light cycle), and fed with sufficient water and food.
Wild animals	The study did not involve wild animals.
Reporting on sex	Male mice were used in this study. Although we have used single-sex animals in our research, we think that the research results were not only applicable to single sex.
Field-collected samples	The study did not involve samples collected from the filed.
Ethics oversight	All animal studies were conducted under the guidelines set by Tianjin Committee of Use and Care of Laboratory Animals, and the overall project protocols were approved by the Animal Ethics Committee of Nankai University.

Note that full information on the approval of the study protocol must also be provided in the manuscript.
